# Protocol to evaluate the antiviral effect of FDA-approved drugs against dengue virus in Huh7 cells and AG129 mice

**DOI:** 10.1016/j.xpro.2024.102992

**Published:** 2024-04-02

**Authors:** Selvin Noé Palacios-Rápalo, Jonathan Hernández-Castillo, Carlos Daniel Cordero-Rivera, Magda Lizbeth Benítez-Vega, Luis Adrián De Jesús-González, José Manuel Reyes-Ruiz, Carlos Noe Farfan-Morales, Juan Fidel Osuna-Ramos, Arely M. Gonzalez-Gonzalez, Raymundo Cruz, Rosa María del Ángel

**Affiliations:** 1 Department of Infectomics and Molecular Pathogenesis, Center for Research and Advanced Studies (CINVESTAV-IPN), Mexico City 07360, Mexico; 2 Unidad de Investigación Biomédica de Zacatecas, Instituto Mexicano del Seguro Social, Zacatecas, Zacatecas, México; 3 Unidad Médica de Alta Especialidad, Hospital de Especialidades No. 14, Centro Médico Nacional “Adolfo Ruiz Cortines”, Instituto Mexicano del Seguro Social (IMSS), Veracruz 91897, México; 4 Facultad de Medicina, Región Veracruz, Universidad Veracruzana (UV), Veracruz 91700, México; 5 Departamento de Ciencias Naturales, Universidad Autónoma Metropolitana (UAM), Unidad Cuajimalpa, Ciudad de México 05348, México; 6 Facultad de Medicina, Universidad Autónoma de Sinaloa, Culiacán 80019, México; 7 Laboratorio de Ingeniería Tisular y Medicina Traslacional, Facultad de Estudios Superiores Iztacala, Universidad Nacional Autónoma de Mexico (UNAM), Mexico City 54090, Mexico

**Keywords:** Cell Biology, Flow Cytometry, Health Sciences, Microbiology, Microscopy, Model Organisms

## Abstract

Finding an effective therapy against diseases caused by flaviviruses remains a challenge. Here, we present a protocol to test Food and Drug Administration-approved drugs that inhibit host nuclear protein import, promoting a reduction of dengue infection. We describe steps for analyzing the drug effect on nuclear import inhibition of cellular and viral proteins by confocal microscopy or western blotting. We then describe procedures for measuring the antiviral drug effects on virus-infected cells by flow cytometry and testing drug efficacy in dengue-infected AG129 mice by survival assays.

For complete details on the use and execution of this protocol, please refer to Palacios-Rápalo et al.^1^

## Highlights

• Protocol for measuring the antiviral effect of FDA-approved drugs on DENV infection
• Procedures for measuring the FDA-approved drugs effect on DENV proteins nuclear import
• Measurement of effect of nuclear import inhibitors on DENV proteins localization *in vivo*

## Abstract (editor highlights)

Publisher’s note: Undertaking any experimental protocol requires adherence to local institutional guidelines for laboratory safety and ethics.

## Graphical abstract

**Figure:**
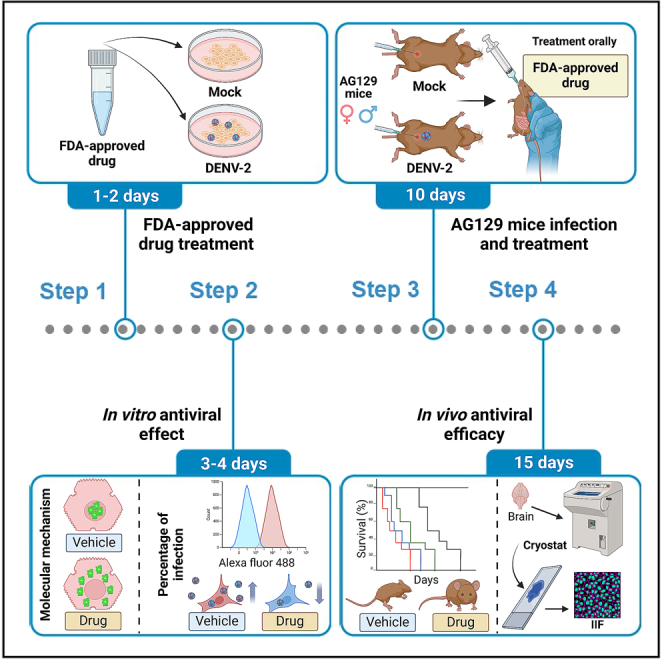

## Before you begin

### Institutional permissions

Obtain animal study approval from your local or institutional Animal Care and Use Committee (CICUAL). This protocol (048–02) was approved by CICUAL at CINVESTAV-IPN, Mexico. The animal experiment followed the Official Mexican Standard Guidelines for Production, Care, and Use of Laboratory Animals (NOM-062-ZOO-1999).

### Preparation of research materials

1. Acquire all critical reagents mentioned in the key resources table.
2. Acquire cell lines to be used in this protocol and prepare enough frozen stocks.
3. Acquire dengue virus (DENV) stock to be used in this protocol.
4. Prepare media using recipes described in this protocol.

**CRITICAL:** All procedures must be performed in a BSL-2 certified laboratory equipped with a biosafety cabinet using standard aseptic techniques. Cultures are grown and maintained in a humidified incubator at 37°C with 5% CO_2_.

### Cell culture

**Timing: 1–2 days**

5. Huh7 Cell Growth.
  a. Culture Huh7 cells in 100 mm plates at 37°C with 5% CO_2_ in a complete DMEM medium.

***Note:*** Cells at 80%–100% confluency are required for this procedure.

6. Obtaining the cell suspension.
  a. Remove the supernatant.
    i. Wash with 5 mL of cold 1X PBS to remove excess medium containing fetal bovine serum (FBS).
  b. Add 4 mL 0.1% trypsin-EDTA.
    i. Incubate for 1–3 min at 25°C until cells are rounded as observed under a microscope.
      ***Note:*** From a 100 mm plate, we expect to have 1 × 10^7^ cells.
  c. Remove the trypsin-EDTA.
    i. Add 5 mL of complete DMEM medium to inactivate the trypsin-EDTA.
      ***Note:*** Gently pipette the cells up and down until a homogeneous suspension is obtained and placed in conical tubes for centrifugation.
  d. Centrifuge at 1,900 × *g* for 7 min at 25°C.
    ***Note:*** Eliminate the clarified supernatant, preserving the integrity of the cell pellet.
  e. Resuspend the cells with 5 mL of DMEM complete medium.
  f. Wash the cells once with 1X PBS. Repeat step d).
    ***Note:*** Depending on the planned experiment, dilute harvested cells to the desired concentration using the DMEM complete medium.

### Electroporation of Huh7 cells

**Timing: 2 days**

This procedure is based on the protocol of Hashemi et al.^2^

7. Preparation of cells prior to transfection.
  a. Starting from the cell suspension of a 100 mm plate at 80% confluence (8 × 10^6^ cells), centrifuge at 1,900 × *g* for 7 min at 25°C.
  b. Discard the clarified supernatant.
    ***Note:*** Ensuring it does not dislodge or disrupt the cell pellet.
  c. Resuspend the cells with 5 mL of cold 1X PBS sterile.
  d. Centrifuge at 1,900 × *g* for 7 min at 25°C.
  e. Repeat step c) twice.
8. Cell Transfection.
  a. Resuspend the cell pellet (8 × 10^6^ cells) with 90 μL of OptiMem.
  b. Add 5 μg of the plasmid to the cell suspension. Resuspend very gently.
  c. Transfer the cells to a 4 mm (electrode spacing) Gene Pulser cuvette.
    **CRITICAL:** Program the Gene Pulser Xcell electroporator (Bio-Rad, Germany) with the following program: 170 V pulse length and 40 ms exponential decay.
  d. Electroporate the cells with the above program.
  e. In a new conical tube, add 10 mL of transfection medium (see materials and equipment setup), and add the transfected cells.
  f. Resuspend the cells very gently.
    **CRITICAL:** The medium should be free of antibiotics.
  g. Transfer 3.4 × 10^5^ cells into 24-well dishes with sterile 14 mm coverslips.
  h. Incubate for 18–24 h at 37°C in CO2 incubator.
  i. Remove the transfection medium.
  j. Add 500 μL of fresh transfection medium supplemented with FDA-approved drugs diluted to the desired test concentration.
    ***Note:*** We chose a drug concentrations that did not reduce the overall cell viability below 80% (compared to untreated cells), as determined using cell viability assays (MTT assays).
  k. Allow treatment for a further 24–48 h.
9. Collection of slides with transfected cells.
  a. Remove the transfection medium.
    i. Wash with 500 μL of cold 1X PBS once in constant agitation at low speed.
  b. Prepare the slides with your transfected cells for indirect immunofluorescence (see section 1: in vitro measurement of the molecular antiviral effect of nuclear import inhibitor drugs).

### RT-qPCR

**Timing: 1–2 days**

10. Sample collection.
  a. Obtain tissues of AG129 mice.
    i. Uninfected (control).
    ii. Infected with DENV-2 and treated with FDA-approved drugs.
      **Pause point:** after obtaining the mice tissues of interest, freeze them on dry ice and store them at −80 C until use. Once you are ready, continue the process from step b).
  b. Add 1 mL of TRIzol to the sample and disaggregate the 100 mg of tissue using a homogenizer (TissueRuptor II, QIAGEN).
  c. Extract total RNA from 2 or 3 mice per condition using the TRIzol reagent manufacturer’s procedure.
    ***Note:*** Each mouse is considered one experimental unit.
  d. Treat samples with DNase I to avoid DNA contamination.
11. Determine the concentration of total RNA using the nanodrop (*Nandodrop 2000*, Thermo Scientific).
12. Perform reverse transcription of 1 μg of total RNA to cDNA using the Moloney Murine Leukemia Virus (M-MLV) reverse transcriptase kit.

**Pause point:** cDNA can be stored at 4°C for 1 day or −20°C for a few weeks. Once you are ready, continue the process from step 13a).

13. Perform quantitative polymerase chain reaction (qPCR).
  a. Use primers corresponding to the C protein region (see key resources table).
  b. For each PCR reaction, use:

**Table:** PCR reaction master mix

Reagent	Amount
DNA template (200 ng)	2 μL
SYBR Green (BIO-RAD)	5 μL
Primer Fw	0.5 μL
Primer Rev	0.5 μL
ddH_2_O	2 μL
**Total**	**10 μL**

    **CRITICAL:** It is important to thoroughly mix the reaction mixture before adding RNA while on ice. Please protect it from light.
  c. Perform a standard curve (10^8^, 10^7^, 10^6^, 10^5^, 10^4^, 10^3^, 10^2^).
    i. From a plasmid possessing a 151 bp insert of the DENV-2 genome corresponding to the previously amplified C protein region.
      ***Note:*** The plasmid containing the 151 bp corresponding to a region of the C protein of DENV was made by Angel-Ambrocio et al.^3^
      ***Note:*** To obtain the initial volume of the stock plasmid, use the following formula:
    ii. Mass of one plasmid molecule.
      To calculate the mass of one plasmid molecule, the following formula is used:
      m=(n) (1.096 × 10^-21^ grams/base pairs).
      m = mass.
      n = number of base pairs (bp) of the plasmid (vector + insert).
      The pJETL2/blunt vector has a total of 2974 bp, and the insert of the C protein region is 151 bp, so the "n" of our plasmid is 3125 bp.
      The mass of 1 plasmid would be (3125 bp) (1.096 × 10^-21^ *g*/bp) = 3.425×10^-21^ *g*/copy.
    iii. Calculation of plasmid copy number.
      To calculate the grams that correspond to the number of copies of interest, the following formula is used:
      g=(#copies) (m).
      To obtain 10^10^ copies, we require:
      (10^10^ copies) (3.425 × 10^-21^ g/copy) = 3.425×10^-7^ grams.
    iv. Establish the standard curve.
      Starting from a 1 μg/μL (1e^-6^ *g*/μL) concentration of plasmid, determine the starting point of the standard curve (example: 10^10^ copies). To obtain the initial volume of the stock plasmid, use the following formula:
      Initial volume= (grams of 10^10^ copies) ÷ (grams/μL of stock plasmid).
    v. Initial volume= (3.425 × 10^-7^grams) ÷ (1 × 10^-6^ g/μL) = 0.34 μL.
      ***Note:*** From this initial volume with 10^10^ copies, perform serial dilutions.
  d. Use the thermal cycling conditions below in the Eco Ilumina System equipment:

**Table:** qPCR cycling conditions

Steps	Temperature	Time	Cycles
Initial Denaturation	95°C	1 min	1
Denaturation	95°C	10 s	35–40 cycles
Annealing/Extension	60°C	30 s	
Hold	4°C	Forever

    ***Note:*** Any equivalent RT-PCR equipment would be good for obtaining the number of viral copies.
14. Data analysis.
  a. Adjust the threshold with a mock-infected mouse sample and the non-templated control.
    ***Note:*** In this protocol, the results were analyzed with EcoStudy software. Any equivalent software would be good for analyzing RT-PCR results.
  b. Express data as viral copies in 100 mg of tissue.
    ***Note:*** To express data as viral copies in 100 mg of tissue, use the following formula:
    i. Copies in 100 mg of tissue= (#copies) (Total RNA) ÷ (cDNA input).
      Copies in 100 mg of infection control: (661 copies) (456.25 μg) ÷ 0.200 μg.
      = 1.5 × 10^6^ copies/100 mg of tissue.

## Key resources table

**Table:** 

REAGENT or RESOURCE	SOURCE	IDENTIFIER
**Antibodies**		

Mouse anti-KPNA1 (1:50)	Santa Cruz	Cat# sc-517105; RRID: AB_2721174
Rabbit anti-NS3 (1:300)	GeneTex	Cat# GTX124252; RRID: AB_11171668
Rabbit anti-NS5 (1:300)	GeneTex	Cat# GTX124253; RRID: AB_11169932
Goat anti-mouse Alexa Fluor 488 (1:750)	Invitrogen	Cat# A21202; RRID: AB_141607
Goat anti-rabbit Alexa Fluor 555 (1:300)	Invitrogen	Cat# A21428; RRID: AB_2535849

**Bacterial and virus strains**		

DENV-2 New Guinea C strain	Kindly donated by Instituto de Diagnóstico y Referencia Epidemiológicos Dr. Manuel Martínez Báez (InDRE), Mexico.	N/A
Chemically competent *E. coli* DH5α	Invitrogen	Cat# 18265-017

**Chemicals, peptides, and recombinant proteins**		

Advanced DMEM	Gibco	Cat# 12491-015
Fetal bovine serum (FBS)	Gibco	Cat# 16000-044
Glutamine	Sigma	Cat# g8540-100G
Penicillin	Sigma	Cat# p7794-100MU
Streptomycin	Gibco	Cat# 11860-038
Amphotericin B	Gibco	Cat# 15290-018
Hank’s balanced salt solution	Gibco	Cat# 24020-117
Ivermectin	Sigma-Aldrich	Cat# I8898-250MG
Saponin	Sigma-Aldrich	Cat# 47036-250G-F
Hoechst	Santa Cruz	Cat# 62249
Paraformaldehyde (PFA)	Sigma-Aldrich	Cat# 158127-500G
Veridex (ivermectin)	Laboratorios Maver de México	N/A
APOTEX (atorvastatin)	Protein S.A. de C.V. México	N/A
VECTASHIELD	Vector Laboratories	Cat# H-1000
Opti-MEM	Gibco	Cat# 11058-021
TRIzol reagent	Invitrogen	Cat# 15596026.PPS
DNase I	New England BioLabs	Cat# M0303
Porcine skin gelatin	60 bloom, Ted Pella Inc	Cat# 19225
Chromium (III) potassium sulfate dodecahydrate	Merck	Cat# 1036
Microscope slides (26 × 76mm)	EUROTUBO slides Deltalab	Cat# D100004
Tissue freezing medium	Leica	Cat# 14020108926
Hydrophobic barrier PAP pen	ImmEdge	Cat# H-4000

**Critical commercial assays**		

3-(4,5- dimethylthiazol-2-yl)-2,5 diphenyltetrazolium bromide (MTT)	Sigma-Aldrich	Cat# M5655-500MG
SYBR Green PCR-master mix	Bio-Rad	Cat# 172-5150
Zippy plasmid miniprep kit	Zymo Research	Cat# D4019
Moloney murine leukemia virus (M-MLV) reverse transcriptase kit	Invitrogen	Cat# 28025-013

**Experimental models: Cell lines**		

Huh-7	Kindly donated by Dr. Rivas from Universidad Autonoma de Nuevo Leon, Mexico.	N/A

**Experimental models: Organisms/strains**		

AG129 mice: strain 129/Sv mice doubly deficient in IFN-α/β and -γ receptors	La Jolla Institute for Immunology	N/A

**Oligonucleotides**		

Primer DENV capsid gene forward: 5′-CAATATGCTGAAACGCGAGA-3′	Prada-Arismendy et al.^4^	N/A
Primer DENV capsid gene reverse: 5′-TGCTGTTGGTGGGATTGTTA-3′	Prada-Arismendy et al.^4^	N/A

**Recombinant DNA**		

SV40 NLS-x4GFP	Kindly donated by Dr. Bulmaro Cisneros from the Department of Genetic and Molecular Biology-CINVESTAV.	N/A

**Software and algorithms**		

Icy image analysis	Chaumont, et al.^5^	https://icy.bioimageanalysis.org
GraphPad Prism 6.0	GraphPad Software, San Diego, California, USA	https://www.graphpad.com
FlowJo v10 software	BD Life Sciences	https://www.flowjo.com/solutions/flowjo
Leica Application Suite X Core v3.3.0	Leica	https://www.leica-microsystems.com/products/microscope-software/details/product/leica-las-x-ls/
EcoStudy software v5.04890	Illumina	Eco Software v5.0 (illumina.com)

**Other**		

TissueRuptor II	QIAGEN	Cat# 9002755

## Materials and equipment

**Table:** DMEM complete medium (Huh7 medium)

Reagent	Final concentration	Amount
DMEM Advance	N/A	889 mL
Glutamine (200 mM)	4 mM	20 mL
Fetal bovine serum (FBS)	7%	70 mL
Amphotericin B (1000X)	1X	1 mL
Penicillin (100X)	50 U/mL	10 mL
Streptomycin (100X)	50 μg/mL	10 mL
**Total**	**N/A**	**1000 mL**

Note: Store at 4°C for up to 3 months

**Table:** Transfection medium

Reagent	Final concentration	Amount
DMEM Advance	N/A	894 mL
FBS	15%	15 mL
Glutamine (200 mM)	4 mM	20 mL
Fetal bovine serum (FBS)	7%	70 mL
Amphotericin B (1000X)	1X	1 mL
**Total**	**N/A**	**1000 mL**

Note: Store at 4°C for up to 3 months.

**Table:** Permeabilization buffer

Reagent	Final concentration	Amount
Saponin	0.2%	0.2 *g*
FBS	1%	0.1 mL
PBS (10X)	1X	99.9 mL
**Total**	**N/A**	**100 mL**

Note: Store at −20°C for a few weeks.

**CRITICAL:** Do not store at RT or 4°C due to easy contamination.

**Table:** Gelatin solution

Reagent	Final concentration	Amount
Porcine skin gelatin	0.5%	0.5 *g*
Chromium (III) potassium sulfate dodecahydrate	0.05%	0.05 *g*
ddH_2_0	N/A	100 mL
**Total**	**N/A**	**100 mL**

Note: Store at 4°C up to 1 month.

## Step-by-step method details

**Figure 1:**
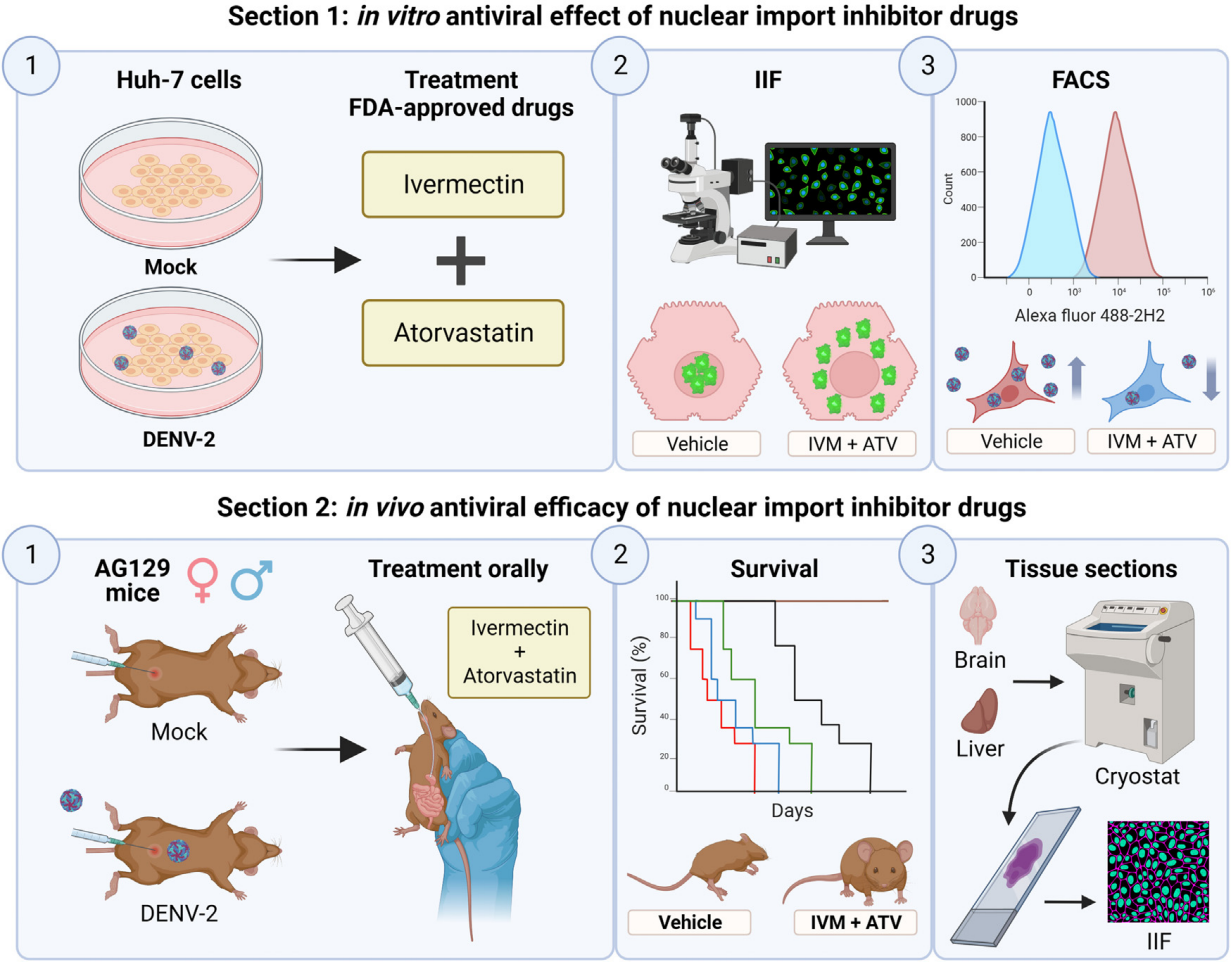
The starting materials are Huh7 cells (*in vitro* assays) and AG19 mice (*in vivo* assays) Section 1: *In vitro* measurement of the molecular antiviral effect of nuclear import inhibitor drugs, IIF to evaluate nuclear import during treatment, and flow cytometry to measure the antiviral effect of nuclear import inhibitor drugs. Section 2: Survival assay to measure the antiviral efficacy of nuclear import inhibitor drugs in DENV-infected AG129 mice and obtaining tissue sections to analyze the nuclear localization of DENV proteins.

This protocol focuses on two sections: 1) the confocal immunofluorescence and flow cytometry approach to measure the antiviral effect of nuclear import inhibitors FDA-approved drugs such as Ivermectin (IVM) or Atorvastatin (ATV) and 2) mice survival assays to measure the antiviral efficacy of these drugs (Figure 1).

### Section 1: *In vitro* measurement of the molecular antiviral effect of nuclear import inhibitor drugs

**Timing: 4–7 days**

This section describes the protocol to measure, in cell monolayers, the effect of nuclear import inhibitor drug treatment on the subcellular localization of host and viral proteins by confocal microscopy or Western blot. In addition, we measured the percentage of DENV-2 infected cells by flow cytometry after treatment with both drugs.

#### Nuclear import inhibitor FDA-approved drug treatment

**Timing: 1–2 days**

The following procedure is described based on Huh7 cells grown in 24-well dishes on coverslips at 70%–80% confluence to analyze the nuclear import of DENV-2 proteins early in the infection during treatment with nuclear import inhibitor FDA-approved drugs. The drugs must be prepared in sterile conditions, aliquoted, and kept at −20°C until use. We used a plasmid constructed with an SV40 NLS (RKRK) tagged with four GFP to validate the drug’s ability to inhibit nuclear import. This NLS is recognized by the importin α/β pathway, which IVM or ATV4 can inhibit.

1. Set up nuclear import inhibition control.
  a. Start from Huh7 cells transfected by electroporation with the plasmid SV40 NLS-x4GFP (see the electroporation of Huh7 cells procedure).
  b. Remove the transfection medium supplemented with the drugs IVM, ATV, combined IVM+ATV, or vehicle for 24 h.
    **CRITICAL:** If you observe loss of monolayers from the coverslips after treatment, see troubleshooting, problem 1.
  c. Add 300 μL of cold 4% paraformaldehyde (PFA) per well and fix the cells at 4°C for 30 min.
    **Pause point:** If samples cannot be processed immediately, replace PFA with 500 μL per well of sterile 1X PBS and keep at 4°C up to a maximum of 1 week. Seal the plate well to avoid drying out. Once you are ready, continue the process from step 3, c).
2. Set up Huh7 cell infection with DENV-2.
  a. Remove the complete DMEM medium.
  b. Add DENV-2 of desired MOI suspended in Hank’s medium.
  c. Allow infection for 2 h at 37°C in CO2 incubation.
    ***Note:*** Use minimally 200 μL of Hank's for 24-well dishes to prevent wells from drying out during infection. Please adjust the volume if you use different culture dishes to perform this assay.
    ***Note:*** The multiplicity of infection (MOI) is calculated depending on the experiment. For early infection assays (8 h or 12 h), use an MOI of 10; for assays longer than 24 h, use an MOI of 0.5 or 1.
  d. Remove Hank’s medium.
  e. Add a complete medium supplemented with drugs or vehicles to mock-infected or infected cells.
  f. Allow infection in the presence of treatment for 12 h for early and 24 h for late infection assays.
    **CRITICAL:** If you observe loss of monolayers from the coverslips after treatment, see troubleshooting in problem 1.
    ***Note:*** Use 500 μL of complete DMEM medium for 24-well dishes. Please adjust the volume if you use different culture dishes to perform this assay.
    ***Note:*** Early and late infection assays allow the evaluation of the nuclear localization of different DENV proteins during the viral replicative cycle.

#### Indirect immunofluorescence (IIF) to evaluate nuclear import during treatment

**Timing: 2 days**

In this step, after drug treatment, antibodies are added sequentially to study the subcellular localization of host nuclear transport receptors (NTR) and DENV proteins that have nuclear localization (non-structural protein 3 and 5).

**Figure 2:**
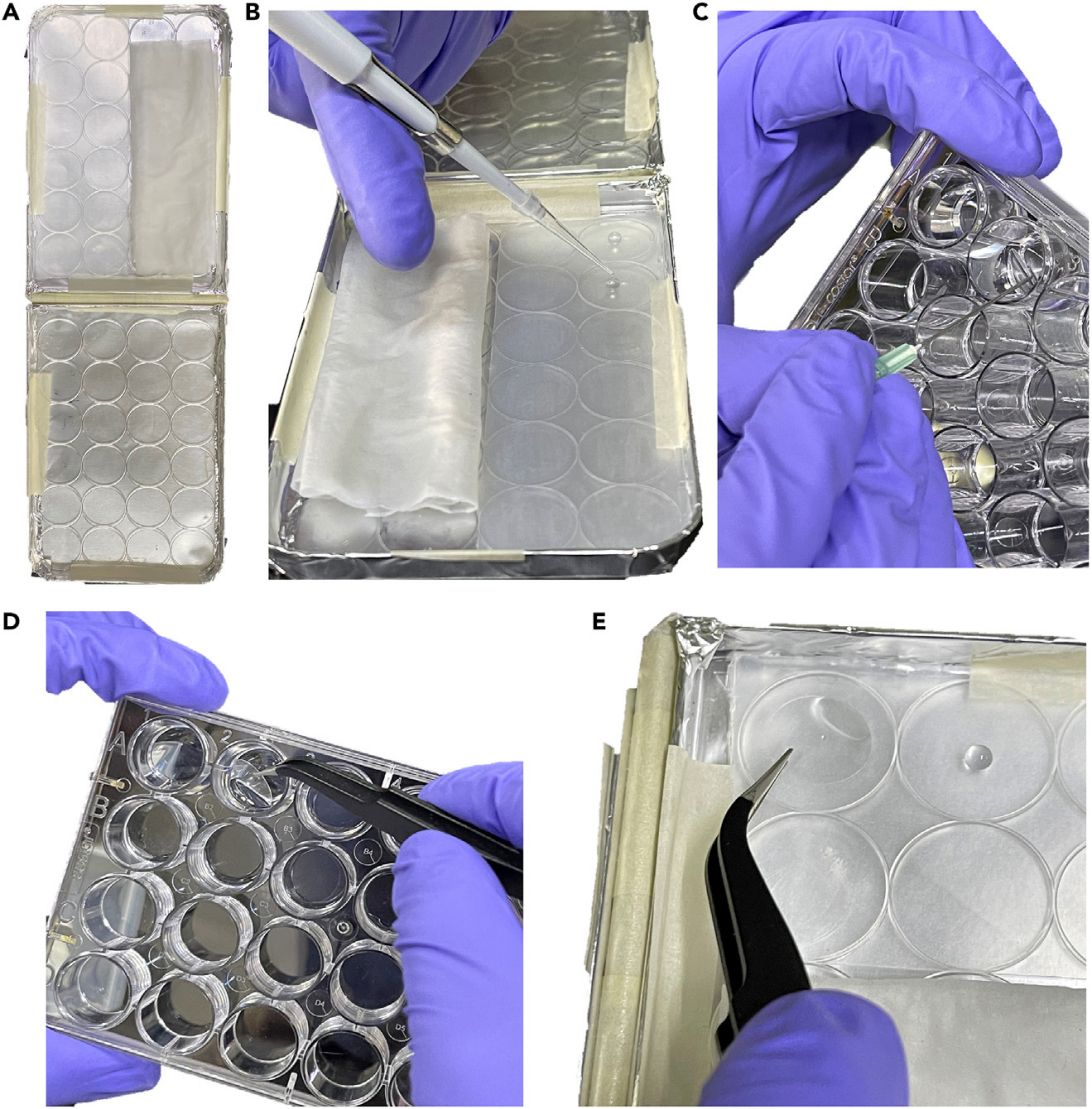
Incubation of primary and secondary antibodies in a humidity chamber (A) Humidity chamber. (B) Drop the antibody in a permeabilizing solution into the center of the well. (C) Lift the coverslip with long-tipped forceps (or a needle with a bent tip). (D) Remove the coverslips with the help of long-tipped forceps. (E) Leave the coverslip on top of the drop (the cell monolayer should touch the drop).

**Figure 3:**
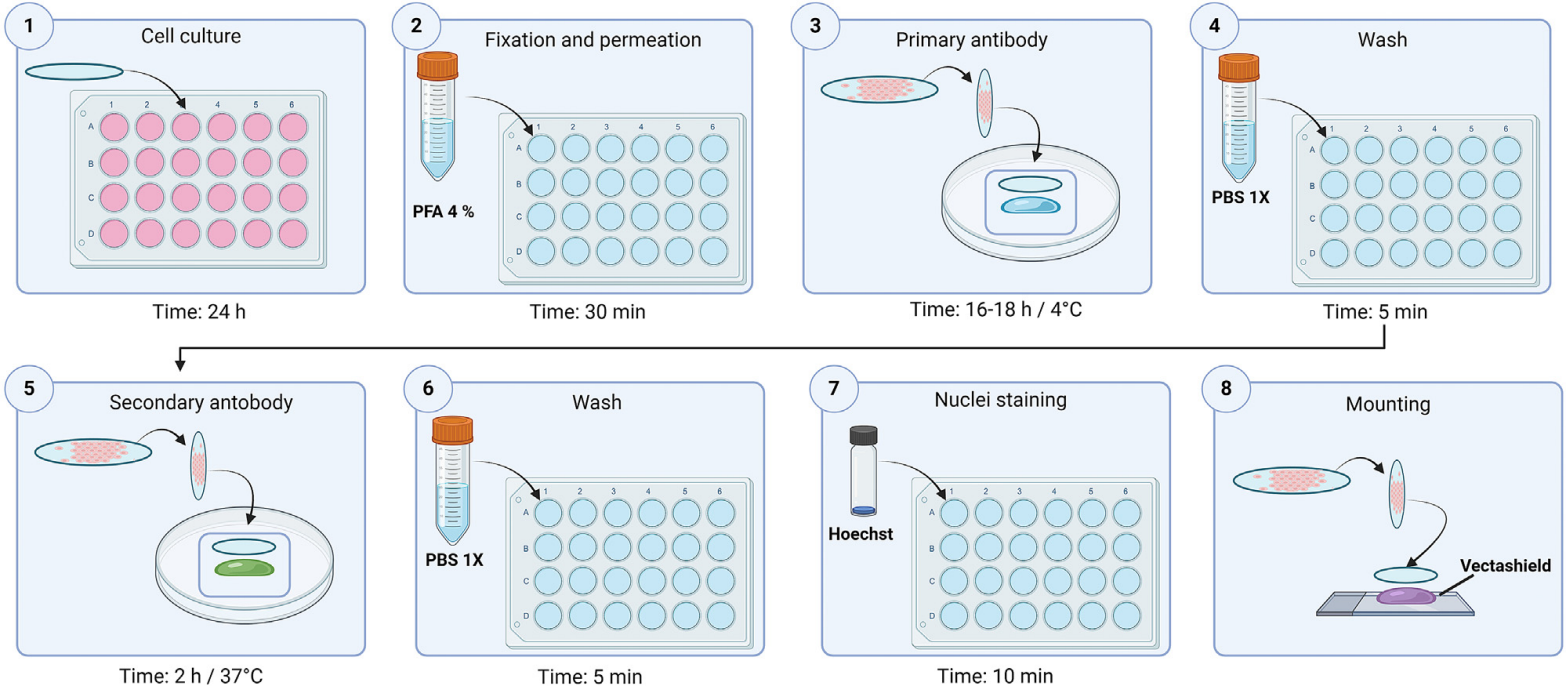
Schematic representation of the IIF assay Step 1: culture cells on coverslips. Step 2: Fix and permeabilize. Step 3: Incubate primary antibody 16–18 h at 4°C. Step 4: Wash with 1X PBS. Step 5: Incubation of secondary antibody for 2 h at 37°C. Step 6: Washes with 1X PBS. Step 7: Nuclear staining with Hoechst. Step 8: Mounting with Vectashield.

3. Set up IIF to analyze nuclear import.
  a. Remove the complete DMEM medium.
  b. Wash once with 500 μL per well of 1X PBS.
  c. Add 300 μL of cold 4% PFA per well and fix the cells at 4°C for 30 min.
    **Pause point:** If samples cannot be processed immediately, replace PFA with 500 μL per well of PBS 1X sterile and keep at 4°C for one week. Seal the plates well to avoid them from drying out. Once you are ready, continue the process from step d).
  d. Remove PFA or 1X PBS from step c).
    i. Wash with 1X PBS three times, each 500 μL per well in slow stirring for 5 min.
  e. For each well, add 300 μL of permeabilization buffer (0.2% saponin with 1% fetal bovine serum) in low agitation for 30 min at 25°C.
    ***Note:*** The permeabilization buffer permeabilizes and blocks non-specificity in the cells prior to labeling with antibodies.
    ***Note:*** Cells transfected with SV40 NLS-containing plasmid do not need to be stained with any antibody; skip to step m).
  f. Prepare mouse anti-KPNA1 (1:50), rabbit anti-NS3 (1:300), or rabbit anti-NS5 (1:300) antibodies in a permeabilization buffer.
  g. In a humidified chamber covered with parafilm, drop 15 μL of antibody per well and leave the coverslip on top (Figure 2).
    **CRITICAL:** To prevent the cells from not staining with the antibody, avoid leaving bubbles when placing the coverslip on top.
  h. Incubate at 4°C (16–18 h). Seal the plate well to avoid drying out.
  i. Return the coverslips to the 24-well plate and wash 3 times with 1X PBS in low agitation for 5 min.
    **Pause point:** If samples cannot be processed immediately, add 500 μL per well of PBS 1X sterile and keep at 4°C for a few days. Seal the plate well to avoid drying out. Once you are ready, continue the process from step j).
  j. Prepare secondary antibodies, anti-mouse Alexa 488 (1:1000) or anti-rabbit Alexa 555 (1:300), in the permeabilization buffer.
  k. In a humidified chamber covered with parafilm, drop 15 μL of secondary antibodies per well and leave the coverslip on top (Figures 2 and 3).
  l. Incubate 2 h at 37°C. Seal the plate well to avoid drying out. Then repeat step i).
    **Pause point:** If samples cannot be processed immediately, add 500 μL per well of PBS 1X sterile and keep at 4°C for a few days. Seal the plate well to avoid drying out and cover it from light. Once you are ready, continue the process from step m).
    **CRITICAL:** From this step, protect the coverslips from light.
  m. Prepare Hoechst’s stain (1:1000) in PBS 1X and add 300 μL per well in low agitation for 10 min. Then repeat step i).
  n. Finally, mount the coverslips with 3 μL of vectashield on clean slides and seal the edges (example: nail varnish) (Figure 3).
    **Pause point:** If the samples cannot be visualized, store the slides protected from light at 4°C for short-term storage or at −20°C for long-term storage.
4. Visualize the samples under a confocal microscope.

**CRITICAL:** If you observe antibodies or the Hoechst mark has a different location than usual, see troubleshooting, problem 2.

***Note:*** We used the Leica TCS SP8 Confocal Microscope (Leica Microsystems) in this protocol. The objective used was 63× oil lens, and the resolution of images is 1024 × 1024 pixels. Any equivalent confocal microscope with similar settings would be suitable for producing confocal images for later quantification analysis.

5. Confocal image analysis.

***Note:*** The images were analyzed with the Leica Application Suite X Core Offline v3.3.0 software in this protocol.

***Note:*** The images obtained were imported into Icy software, and we determined the mean fluorescence intensity (MFI) in the regions of interest (ROI) of at least 30 cells per condition.

#### Flow cytometry to measure the antiviral effect of nuclear import inhibitor drugs

**Timing: 2 days**

This step is optimized for the analysis of the percentage of infection of Huh7 cells infected with DENV2 treated with IVM, ATV, combined IVM+ATV, or Vehicle by flow cytometry. These cells are seeded in 12-well plates at 70%–80% confluency.

6. Obtaining the cell suspension.
  a. Remove the supernatant.
  b. Wash with 1 mL of cold 1X PBS to remove excess medium containing FBS.
  c. Add 500 μL of 0.1% trypsin-EDTA and incubate for 1–3 min at RT until cells are rounded.
    ***Note:*** Supernatants can be stored at −80°C for plaque assay or other forms of analysis as desired by the user.
  d. Immediately remove the trypsin-EDTA.
  e. Add 1 mL of complete DMEM medium to inactivate the trypsin-EDTA.
    i. Gently pipette the cells up and down until a homogeneous suspension is obtained, then place them in a new 1.5 mL tube.
  f. Centrifuge at 50 × *g* for 8 min at 4°C.
  g. Eliminate the clarified supernatant, preserving the integrity of the cell pellet.
  h. Resuspend the cells with 200 μL of 1X PBS.
  i. Subsequently, add 600 μL of cold 4% paraformaldehyde (PFA) for 30 min under constant agitation at RT.
    **CRITICAL:** Allow good cell homogenization before adding the fixation solution. Poor homogenization of single cells leads to the fixation of cell clumps, favoring cell rupture and formation of cell debris. It can be recognized as double events in the flow cytometer.
    **CRITICAL:** If you have problems with this step, please go to the troubleshooting section in problem 3.
  j. Add 200 μL of 1X PBS and centrifuge at 500 × *g* for 8 min at 4°C.
  k. Remove the clarified supernatant, preserving the integrity of the cell pellet.
  l. Add 1 mL of 1X PBS. Resuspend gently.
  m. Repeat step j) and resuspend in 300 μL of 1X PBS.
    **Pause point:** Cells in 1X PBS can be stored for further analysis. Store the cells at 4°C for future analysis for 1–2 weeks maximum. Once you are ready, continue the process from step 7a).
    **CRITICAL:** Do not store at −80°C.
7. Set up cell labeling.
  a. Add 1 mL of 1X PBS and centrifuge at 500 × *g* for 8 min at 4°C.
  b. Remove the clarified supernatant, preserving the integrity of the cell pellet.
  c. Add 500 μL of permeabilizing solution.
  d. Resuspend the cells and incubate for 30 min under constant agitation at RT.
  e. Add 1 mL of 1X PBS and centrifuge at 500 × *g* for 8 min at 4°C.
  f. Remove the clarified supernatant, preserving the integrity of the cell pellet.
  g. Add 50 μL of 2H2 antibody (this antibody recognizes the prM protein and part of the E protein) (1:50) in a permeabilizing solution.
  h. Incubate at 4°C under constant agitation.
  i. Add 1 mL of 1X PBS and centrifuge at 500 × *g* for 8 min at 4°C.
  j. Remove the clarified supernatant, preserving the integrity of the cell pellet.
  k. Add 50 μL of Alexa 488 secondary antibody (1:750) in a permeabilizing solution.
  l. Incubate for 2 h at 25°C with constant agitation.
    ***Note:*** The sample should be protected from light due to the fluorescence of the secondary antibody.
    **CRITICAL:** The cells must be constantly stirred to ensure the primary and secondary antibodies are correctly incorporated. However, it is possible that cells may remain associated with the tube walls, so it is essential to recover as many cells as possible by detaching with 1X PBS solution.
  m. Add 1 mL of 1X PBS and centrifuge at 500 × *g* for 8 min at 4°C.
  n. Remove the clarified supernatant, preserving the integrity of the cell pellet.
  o. Add 300 μL of 1X PBS.
    **Pause point:** Cells in 1X PBS can be stored for further analysis. Store the cells at 4°C for future analysis for 1–2 weeks maximum.
8. Analyze the samples in the flow cytometer.

***Note:*** In this protocol, we use the BD LSR Fortessa. The parameters used for sample analysis were as follows. A Threshold of 15,000 was used, and voltages of FSC (240 V), SSC (260 V), Alexa Flour 488 (375 V), and Pacific Blue (320 V) were used to determine cell autofluorescence. Any equivalent flow cytometer with similar settings should be suitable to produce data for later quantification analysis.

9. Cytometry data analysis.

***Note:*** The data were analyzed using FlowJo v10 software. The total population (SSC-A vs. FSC-A) and single events (FSC-H vs FSC-A) were adjusted by analyzing 10,000 events.

### Section 2: Survival assay to measure the antiviral efficacy of nuclear import inhibitor drugs in DENV-infected AG129 mice

**Timing: 4–8 weeks**

This section describes the protocol to test nuclear import inhibitor FDA-approved drugs in DENV-2-infected AG129 mice by survival assays and the procedure to obtain tissue sections to analyze the nuclear localization of viral proteins *in vivo*.

***Note:*** We use six- to eight-week-old female and male AG129 mice (La Jolla Institute for Allergy and Immunology, Strain: 129/Sv).

#### Survival assay to measure the antiviral efficacy of nuclear import inhibitors

**Timing: 25 days**

10. Determine treatment groups.
  a. Group three female or three male mice for each treatment group. The groups are:
    i. Control (uninfected and untreated mice).
    ii. Vehicle (infected mice treated with water).
    iii. Independent treatment (infected and treated with IVM or ATV).
    iv. Combined treatment (infected and treated with IVM+ATV).

***Note:*** In this protocol, we used 0.6 mg/kg/day of IVM and 20 mg/kg/day of ATV.

11. Set up AG129 mice infection with DENV-2.
  a. Measure the initial weight of each mouse.
  b. Using an insulin syringe, inoculate DENV-2 intraperitoneally in 100 μL of sterile 1X PBS.
  c. Allow infection for 4 days.

***Note:*** For the control group, inoculate only sterile 1X PBS.

***Note:*** AG129 mice were DENV-2 infected with 4 × 107 PFU/mL per mouse in this protocol.

**CRITICAL:** Make sure not to puncture any mouse organ during the injection of the virus.

12. Measure the mice’s weight and clinical score daily (see Table 1).

**Table 1:** Morbidity scale in AG129 mice

Score	Clinical signs of DENV disease
**1**	Healthy.
**2**	Mild signs of lethargy.
**3**	Lethargy, bristly hair, and stooped posture.
**4**	Lethargy, bristly hair, stooped posture, muscle weakness, reduced mobility, and poor stimuli response.
**5**	Moribund: Lethargic, very bristly hair, not able to stand up, no mobility, and no response to stimuli.

Adapted from (Palacios-Rápalo et al.^1^).

**CRITICAL:** Exclude mice that die from causes other than infection.

13. Set up the treatment scheme for AG129 mice.
  a. Prepare commercial drugs (IVM or ATV) for humans in sterile water for mice.
  b. Four days post-infection, using a gastric tube, administer 50–60 μL of the vehicle or treatment orally to simulate the treatment used in humans.

**Figure 4:**
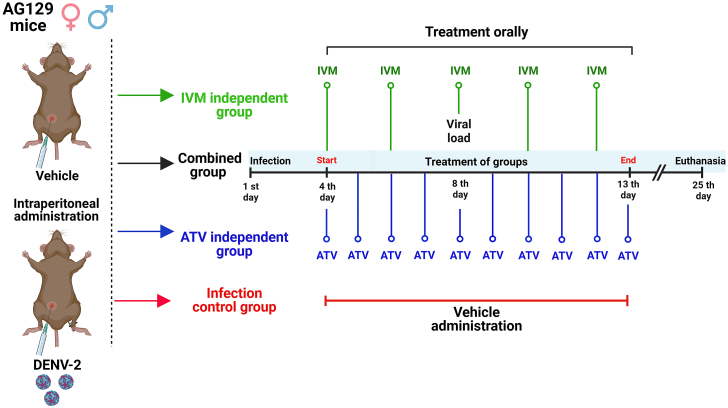
Treatment scheme for testing FDA-approved drugs in DENV-2-infected AG129 mice

**CRITICAL:** Based on the initial weight of each mouse, the concentrations of each drug should be prepared in sterile water in accordance with the doses chosen.

***Note:*** For this protocol, mice were treated with ten doses of ATV once per day and with 5 doses of IVM every other day (see Figure 4).

14. Monitor the development of the disease during and after treatment.
  a. Treatment will end 13 days post-infection, and surviving mice will be euthanized until day 25 (12 days after the end of treatment).
    **CRITICAL:** If you have problems with this step, please go to the troubleshooting section in problem 4.
  b. Measure each mouse’s weight and clinical score (see Table 1) during and after treatment.
    **CRITICAL:** Exclude mice that die from causes other than infection.

#### Obtaining tissue sections to analyze the nuclear localization of DENV proteins

**Timing: 4 days**

15. Preparation of gelatinized slides
  a. In a glass flask, dissolve 0.5% of porcine skin gelatin in distilled water at 60°C with gentle stirring in a stirrer/hot plate.
    ***Note:*** Use a clean thermometer inside the flask to monitor temperature.
    ***Note:*** Gelatin solution can be stored for no more than 1 month at 4°C or before if contamination or debris are not detected.
  b. Once the gelatin is completely dissolved, remove it from the hot plate.
  c. When it reaches approximately 30°C, add 0.05% Chromium (III) potassium sulfate dodecahydrate until dissolved with gentle stirring.
  d. Transfer the gelatin solution to a glass Coplin jar.
    **Pause point:** Store the gelatin solution at 4°C until use. Once you are ready, continue the process from step e).
  e. Place the clean and degreased microscope slides in the Coplin jar with the gelatin solution.
  f. Incubate for 1 min.
    i. Drain the gelatin excess, and place them in the stainless-steel basket.
      **CRITICAL:** The microscope slides must be perfectly clean and degreased for the proper adhesion of the gelatin.
      **CRITICAL:** The gelatin should be at RT prior to use.
  g. Gelatinized slides placed in the basket are covered with aluminum foil to protect them from dust.
    ***Note:*** Incubate gelatinized slides 16–18 h at 37°C to dry.
  h. Store gelatinized slides in a microscope slide holder box at 25°C until use.
    ***Note:*** Prepare only the necessary slides for the complete experiment.
16. Histological Processing.
  a. Immediately after obtaining the mice tissues of interest:
    i. Freeze them on dry ice.
    ii. Cover them with parafilm and aluminum foil.
    iii. Store them at −80 C until use.
      ***Note:*** In this protocol, brains and livers were obtained from DENV-2-infected AG129 mice treated with IVM, ATV, or IVM+ATV.
      ***Note:*** Stored mouse tissues can be analyzed by RT-qPCR to quantify DENV RNA copy number (see RT-qPCR procedure).
      ***Note:*** Pre-fixed tissues (i.e., 4% PFA solution) can also be processed in the same way but must be cryoprotected with a 10% sucrose PBS solution for 24 h at 4°C before freezing.
  b. Adhere the frozen tissue to the cryostat sample holder with a tissue freezing medium.
    i. Align tissue samples to cut in the axis of interest: sagittal, coronal, or transversal (axial).
      **CRITICAL:** Avoid tissue thaw/freeze cycles.
  c. In cryostat (Leica CM1100 with low profile blades, Leica Microsystems), obtain 8 μm thick slices and mount them into gelatin-coated slides.
  d. Store the slides in a holder box at −20°C until use.
17. Tissue immunofluorescence preparation.
  a. Before fixing the tissue sections:
    i. Surround the tissue with a hydrophobic barrier pen to prevent the antibodies from diffusing throughout the slide.
  b. Transfer the slides to a Coplin jar.
    i. fix tissue sections using 4% PFA for 30 min.
  c. Wash three times with 1X PBS.
    **CRITICAL:** If the tissue section detaches from the slice, please see the troubleshooting section in problem 5.
  d. Transfer the slides to a Coplin jar with the permeabilizing solution for 30 min.
  e. Prepare the primary antibody (anti-NS3) in the permeabilizing solution.
  f. Place the slides in a humid chamber.
    i. Add 30–50 μL of primary antibody on each tissue section.
  g. Incubate 16–18 h at 4°C.
  h. Wash three times with 1X PBS.
  i. Prepare the secondary antibody (Alexa Fluor 555) in the permeabilizing solution.
  j. Place the slides in a humid chamber.
    i. Add 30–50 μL of secondary antibody to each tissue section.
  k. Incubate at 37°C for 2 h. Then repeat step h).
    **CRITICAL:** From this step, protect the slides from light.
  l. Prepare Hoechst’s stain (1:1000) in PBS 1X and cover the tissue sections. Then, repeat step h).
  m. Finally, mount the tissue sections with vectashield.
    i. Seal the edges (for example, nail varnish).
      **Pause point:** If the samples cannot be observed, store the slides protected from light at 4°C for short-term storage or at −20°C for long-term storage.
18. Visualize the samples under a confocal microscope.

***Note:*** We used the Leica TCS SP8 Confocal Microscope (Leica Microsystems) in this protocol. The objective used was 63× oil lens, and the resolution of images is 1024 × 1024 pixels. Any equivalent confocal microscope with similar settings would be good for producing confocal images for later quantification analysis.

19. Confocal image analysis.

***Note:*** The images were analyzed with the Leica Application Suite X Core Offline v3.3.0 software.

***Note:*** The images obtained were imported into Icy software and we determined the mean fluorescence intensity (MFI) in the regions of interest (ROI) of at least 30 cells per condition.

## Expected outcomes

### Measurement of nuclear import during IVM or ATV treatment by IIF assay

**Figure 5:**
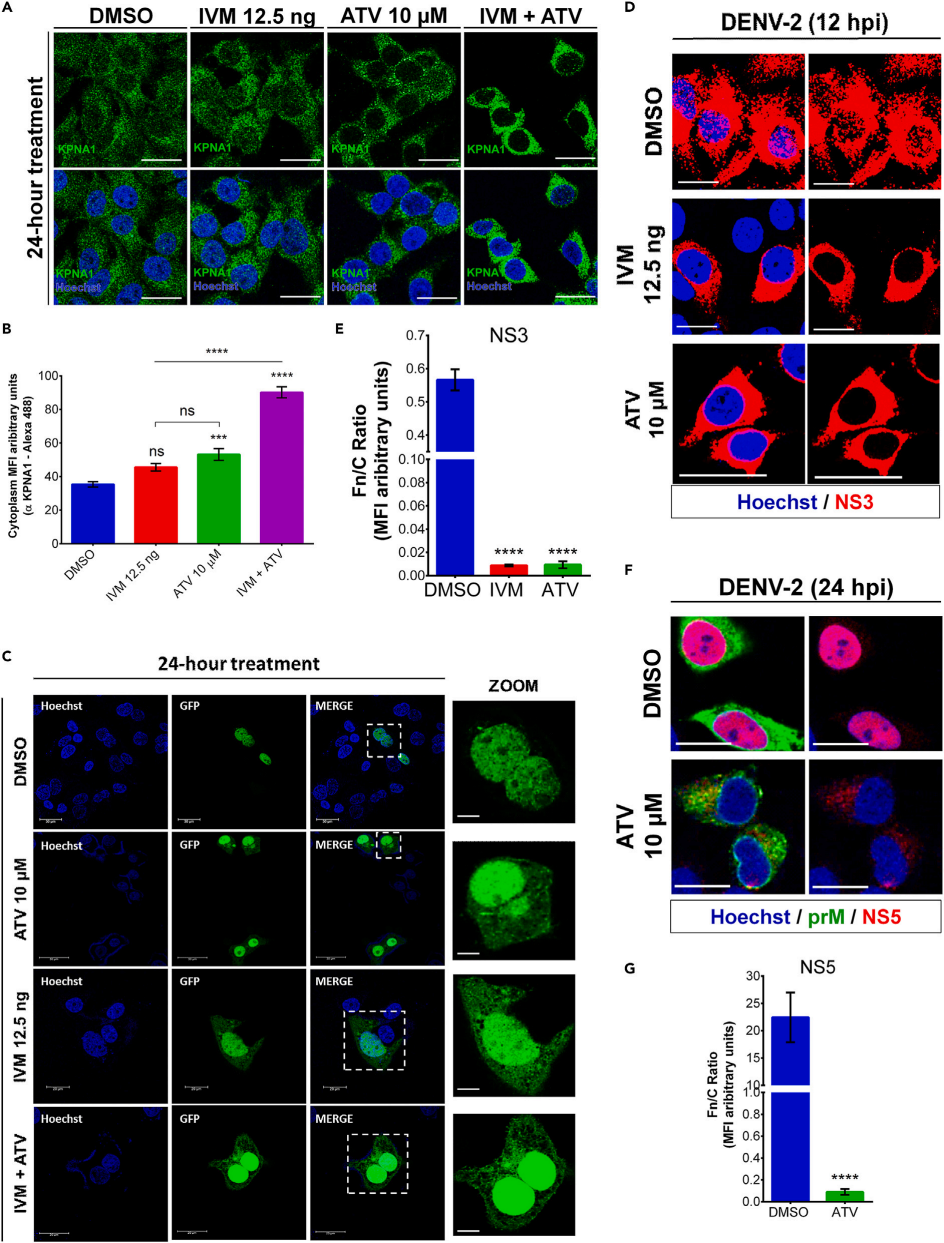
*In vitro* antiviral mechanism of FDA-approved nuclear import inhibitor drugs (A) Huh7 cells treated with vehicle, IVM, ATV, or the combination of IVM+ATV were stained with anti-KPNA1 as a control for drug activity to inhibit nuclear import. Cells were analyzed 24 h post-treatment. Scale bar: 30 μm. (B) Quantification of the cytoplasmic distribution of KPNA1 signal in Huh7 cells at the indicated conditions. n = 30 cells from triplicates. ns, not significant; ∗∗∗p < 0.001; ∗∗∗∗p < 0.0001. (C) Huh7 cells transfected with SV40 NLS-containing plasmid were treated with vehicle, IVM, ATV, or the combination of IVM+ATV as a control for inhibiting nuclear importin α/β pathway-dependent cargo nuclear import. (D) Huh7 cells infected with DENV-2 treated with vehicle, IVM, or ATV were stained with anti-NS3 to assess the drugs' effect on the viral protein’s nuclear localization at 12 hpi. Scale bar: 30 μm. (E) Quantification of the NS3 distribution signal. The nucleus-cytoplasmic fluorescence ratio was used to show the distribution of NS3 protein at the indicated conditions. n = 30 cells from triplicates. ∗∗∗∗p < 0.0001. (F) Huh7 cells infected with DENV-2 treated with vehicle or ATV were stained with anti-NS5 to assess the effect of the drugs on the nuclear localization of the viral protein at 24 hpi. Scale bar: 30 μm. (G) Quantification of the NS5 distribution signal. The nucleus-cytoplasmic fluorescence ratio was used to show the distribution of NS5 protein at the indicated conditions. n = 30 cells from triplicates. ∗∗∗∗p < 0.0001. Figure reprinted with permission from Palacios-Rápalo et al.^1^

The subcellular distribution of cellular and DENV-2 protein during treatment with FDA-approved drugs such as IVM and ATV, which affect the nuclear import of viral and host cellular proteins,^1^ was analyzed by confocal microscopy. Nuclear import of the host cellular protein KPNA1 and NS3 or NS5 proteins of DENV-2 was measured by fluorescence nucleus-cytoplasm ratio (Fn/C). The nuclear dye Hoechst was used to define nuclei, commercial anti-KPNA1 antibody was used to identify NTR of the host cell, and anti-NS3 or anti-NS5 antibodies were used to identify NS3 and NS5 proteins of DENV-2 (Figures 5A–5D and 5F). In mock-infected cells treated with the vehicle, KPNA1 protein was distributed in both the nucleus and cytoplasm compartments. In contrast, in mock-infected cells treated with IVM, ATV, or the combination of IVM+ATV, the distribution of the KPNA1 protein was mainly cytoplasmic (Figure 5A). The same effect was observed with NS3 and NS5 viral proteins; in the presence of a vehicle, the viral proteins were mainly located in the nucleus; in contrast, in the presence of IVM or ATV, the viral proteins were detected mainly in the cytoplasm (Figures 5D and 5F). The ability of IVM or ATV drugs to inhibit nuclear import was confirmed by the partial relocation of the GFP tag to the cytoplasm of cells transfected with the SV40 NLS since in vehicle-treated transfected cells; the GFP tag was abundantly in the nucleus of the cells (Figure 5C). Image analysis and quantification of the data showed that the Fn/C ratio was significantly lower in cells mock-infected or DENV-2-infected treated with IVM or ATV (Figures 5B–5E, and 5G), suggesting that the nuclear import of Huh7 cells was impaired by treatment with these drugs.

### Measurement of the anti-DENV effect of nuclear import inhibitor drugs by flow cytometry

**Figure 6:**
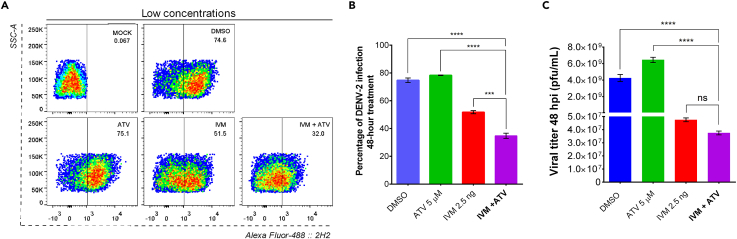
Antiviral effect of FDA-approved nuclear import inhibitor drugs on the percentage of infected cells and DENV-2 viral titer (A and B) The percentage of DENV-2 infection at 48 h was analyzed by flow cytometry from three independent experiments in duplicate. The analyzed population evaluates 10,000 events from the total population’s single events (SSC-H vs. SSC-A). The events are represented as SSC-A vs. Alexa Fluor 488::2H2. ns, not significant; ∗∗∗p < 0.001; ∗∗∗∗p < 0.0001. (C) The viral titer of supernatants from DENV-2 infected cells was determined by plaque assay from three independent experiments. ns, not significant; ∗∗p < 0.01; ∗∗∗∗p < 0.0001. Figure reprinted with permission from Palacios-Rápalo et al.^1^

Using this protocol, we also measured the *in vitro* antiviral effect of treatments with IVM, ATV, and the combination of IVM+ATV1 against DENV. We found that the IVM+ATV combination reduced the percentage of DENV-2 infection more than the individual treatments (Figure 6A). These studies demonstrated that low concentrations of the IVM+ATV combination for 48 h could reduce the percentage of DENV-2 infection in Huh7 cells and viral titer (Figures 6B and 6C), suggesting that we can decrease the toxicity of these drugs by using low concentrations of them.

### Evaluation of antiviral efficacy of nuclear import inhibitor drugs by mice survival assays

**Figure 7:**
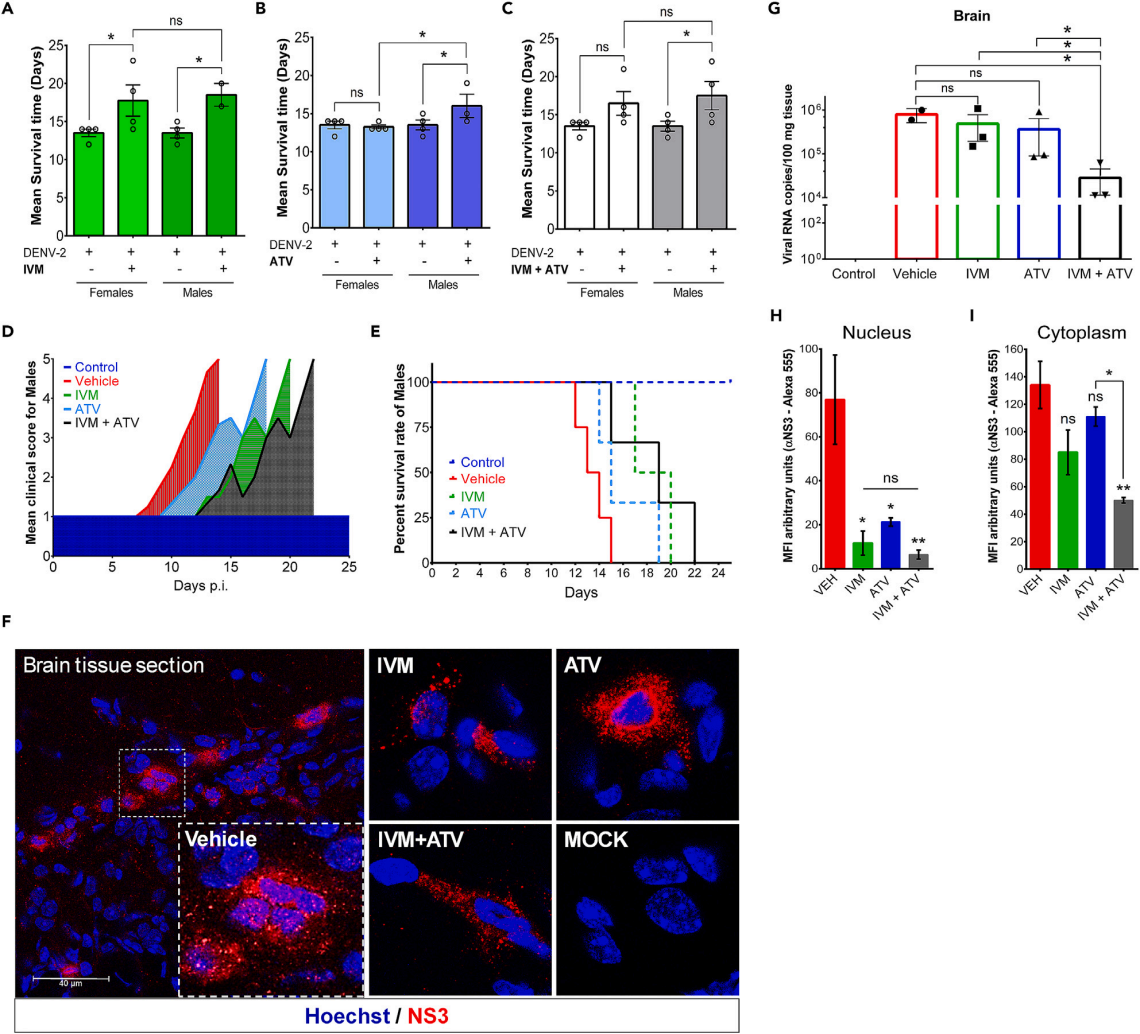
*In vivo* antiviral effect of FDA-approved nuclear import inhibitor drugs (A–C) Mean survival time of AG129 mice treated with IVM, ATV, and the combination IVM+ATV (n = 6 (2 ♂, 4 ♀), 7 (3 ♂, 4 ♀) and 8 (4 ♂, 4 ♀), respectively). Vehicle-treated mice infected with DENV-2 were used as controls (n = 8 (4 ♂, 4 ♀)). ns, not significant; ∗p 0.05. (D) Mean clinical scores of AG129 male mice infected with DENV-2 treated with Vehicle, IVM, ATV, and IVM+ATV. The morbidity scale is shown in Table 1. (E) Kaplan-Meier survival curves represent the percentage survival of male AG129 mice treated with Vehicle, IVM, ATV, and IVM+ATV. The continuous line highlights the treatment that most increased the survival rate compared to the Vehicle. (F) Brain tissue sections from AG129 male mice. Mice received half of the treatment schedule (five days of treatment). Frozen tissue sections were prepared and labeled with anti-NS3 and Hoechst (nuclei). Scale bar: 40 μm. (G) Effect of IVM, ATV, and IVM+ATV treatments on viral load in brains of DENV-2 infected AG129 mice eight days post-infection (n = 3, for each group) compared to vehicle-treated mice (n = 2). Treatment started 4 days post-infection. The independent treated groups received three doses of IVM and five doses of ATV, and the combined group received three doses of IVM+ATV every other day. ns, not significant; ∗p < 0.05. (H and I) Quantification of the NS3 distribution signal. The nucleus-cytoplasmic fluorescence ratio was used to show the distribution of NS3 protein at the indicated condition. The Mean Fluorescence Intensity (MFI) was determined for selected regions of interest (ROIs) in the nucleus and cytoplasm from mice-infected cells of the indicated condition. n = 30 cells. ns, not significant; ∗p 0.05; ∗∗p < 0.01. Figure reprinted with permission from Palacios-Rápalo et al.^1^

AG129 mouse is a lethal model for DENV infection, especially because they are knockout mice for interferon receptors (IFNα/β/γ R−/−),^6^ making them an ideal model for the study of drugs with antiviral activity extending mouse survival. In AG129 mice infected with DENV-2 and treated with the vehicle, the mean survival time is 13–14 days (in both females and males)^1^ (Figures 7A and 7B). These studies showed that mice treated with the IVM+ATV combination extended the mean survival time up to 18 days (Figure 7C), delaying the onset of signs of dengue disease (Figure 7D). Kaplan-Meier plots showed a median survival rate of 19 days in mice treated with the IVM+ATV combination, 6 days longer than mice treated with the vehicle (median 13 days) (Figure 7E). By IIF of tissue sections we observed that exposure to IVM and ATV drugs caused a significant reduction of the presence of NS3 protein in the nucleus (Figures 7F–7H and 7I) and by RT-qPCR a reduction of viral load in the brain of DENV-2-infected AG129 mice was determined (Figure 7G), suggesting that these drugs impair the nuclear import of viral proteins in mouse tissues affecting dengue virus infection.

## Quantification and statistical analysis

### Statistical considerations

Images obtained by confocal microscopy were analyzed using the Icy image analysis software to obtain the mean fluorescence intensity to quantify the Fn/C ratio. The student t-test was used to compare between groups. Flow cytometry data were analyzed using the FlowJo v. 10 software. The ordinary one-way ANOVA with Tukey multiple comparisons *post hoc* test was used to determine significant differences among means of each condition.

### Statistical analysis

For *in vitro* and *in vivo* studies, experimental data are presented as the mean ± standard error of the mean (SEM). For *in vivo* assays, Wilcoxon and Mantel-Cox tests were used to compare the survival rate between treated and untreated groups, and the ANOVA-LSD test was used to compare the average survival time between treated and untreated groups. All statistical analysis was performed using the GraphPad Prism 6.0 software. Differences were considered statistically significant when P values were less than 0.05.

## Limitations

This protocol includes steps and details of methods developed for the study of treatment with IVM and ATV on the nuclear transport of proteins, replication, and pathogenesis of dengue virus *in vitro* and AG129 mice. This standardized protocol for FDA-approved drug analysis against DENV is intended to provide a basis for wider use in studies, including both observational studies and randomized clinical trials. We believe that this protocol will be useful for research laboratories worldwide. Although the protocol works well for us, it can easily be adjusted and improved among the scientific community to the same objective. The effect of nuclear import inhibitory FDA-approved drugs during viral infection has been extensively evaluated in *in vitro* studies. However, one of the limitations of this protocol was to analyze the effect of nuclear import inhibitor drugs on the nuclear transport of DENV proteins in a mouse model due to the difficulty of controlling the time and number of infected cells in the tissues, addition to the fact that there are few methodologies to evaluate nuclear transport in murine models. Furthermore, it is important to note that this protocol was performed in AG129 mice in an immunodeficient murine model, so if it is performed in an immunocompetent murine model, the treatment scheme would have to be readjusted because these mice resolve virus infections. Not every aspect of the protocol has been optimized in detail, and we provided comments and notes whenever other researchers recommended further optimizations and testing.

## Troubleshooting

### Problem 1

Monolayer loss from coverslips after treatment in IIF or flow cytometry assays.

Some drugs, such as ATV (statins), affect the cytoskeleton distribution of treated cells, affecting cell morphology and favoring the loss of the monolayer easily.

### Potential solution

• To avoid detachment of the cells in the monolayer during washes, slowly add 1X PBS against the wall of the well. Carefully perform this step before fixing the cells with 4% PFA in IIF assays.
• During trypsinization, to obtain cells for flow cytometry, remove trypsin quickly (less than 1 min) to avoid damaging cells and losing cells from the monolayer.

### Problem 2

Antibodies or Hoechst stains have other locations than usual.

During handling and marking of slides, there may be a misplaced mark due to a high concentration of salts or bacterial contamination. The antibody concentration and permeabilization solution were not prepared correctly. On the other hand, it may be due to lack of washing.

### Potential solution

• The 1X PBS used to prepare the solutions used in the methodology should be filtered to avoid the appearance of a misplaced mark. Excess salts can cause noise in the images. Increasing the number of washes caring for the cell monolayer can help reduce the image noise.

### Problem 3

Low performance of cell collection for flow cytometry analysis.

Appropriate flow cytometry results are obtained with good cell collection efficiency. Cell aggregates or cell loss may prevent proper analysis of the results.

### Potential solution

• Allow good cell homogenization before adding the fixation solution. Poor homogenization before adding the paraformaldehyde leads to the fixation of cell clumps, favoring cell rupture and formation of cell debris, and can be recognized as double events in the flow cytometer, decreasing single events.
• During manipulation, some cells may adhere to the tube walls. Recover as many cells as possible by detachment with 1X PBS.

### Problem 4

Treatments accelerate disease symptoms in DENV-2-infected AG129 mice.

The high toxicity of some drugs may affect the survival of infected mice, resulting in a lower mean survival time compared to untreated infected mice.

### Potential solution

**Figure 8:**
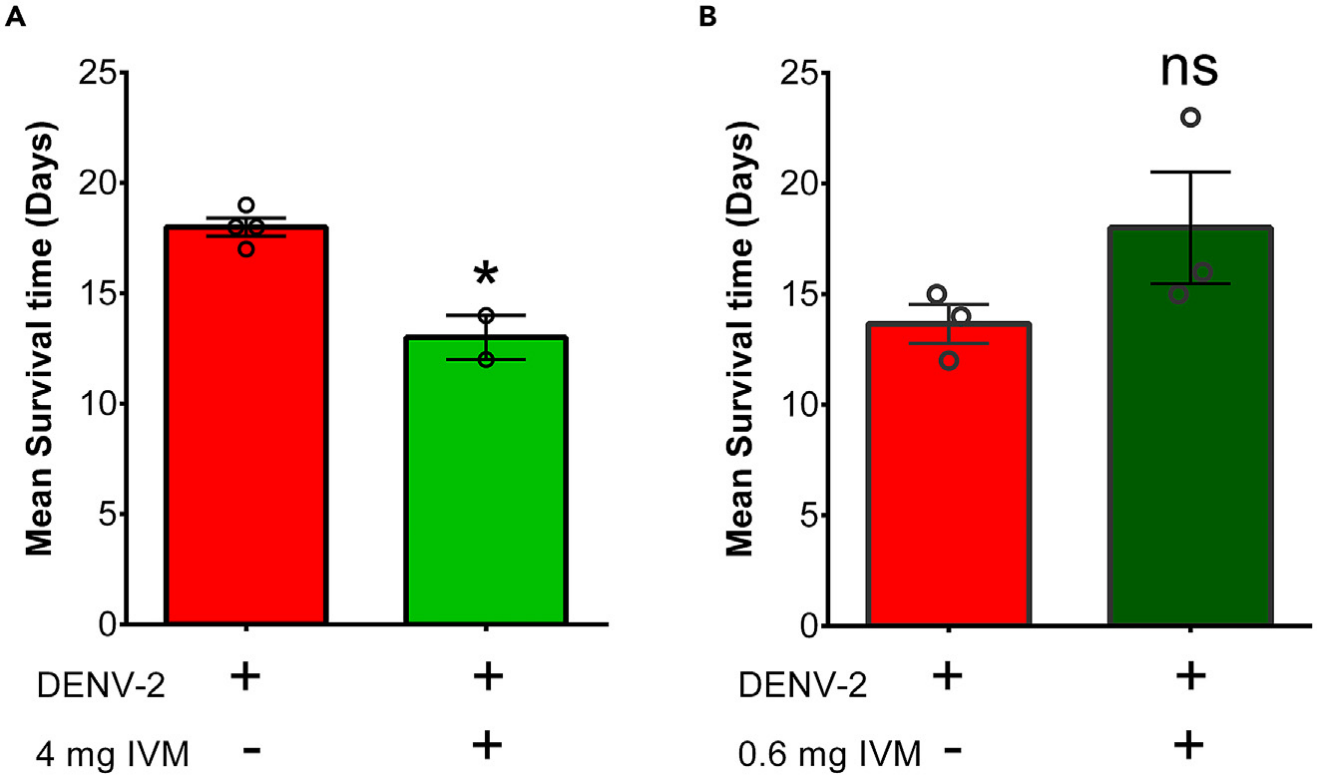
A bad dose of treatment and a good dose of treatment (A) High doses of IVM drug during a 7-day treatment affect the survival time of AG129 mice infected with DENV-2 compared to the vehicle-treated control. ∗p 0.05. (B) Low doses of IVM every other day during a 10-day treatment did not reduce the survival time of AG129 mice infected with DENV-2. ns, not significant. Figure reprinted with permission from Palacios-Rápalo et al.^1^

• To avoid accelerated drug-induced death of DENV-infected mice, decrease the drug concentration and administer one dose every other day. This will reduce the cellular toxicity of the drugs while preserving the antiviral activity (see Figure 8 as an example).

### Problem 5

Detachment of tissue sections from gelatinized slides during the immunofluorescence staining process.

Usually, during fixation or washings, tissue sections are detached from the slide.

### Potential solution

To avoid detachment of tissue sections, degrease and clean the slides properly.

• Immerse the slides in a 2N NaOH solution for 2 h at room temperature in a Coplin jar.
• Rinse thoroughly with running tap water approximately 10 times.
• Rinse thoroughly with distilled water approximately 10 times.
• Place the slides in 100% ethanol for 30 min.
• Air dry the slides at room temperature, protect them from dust, and proceed with gelatinization.

## Resource availability

### Lead contact

Further information and requests for resources and reagents should be directed to and will be fulfilled by the lead contact, Rosa María del Ángel (rmangel@cinvestav.mx).

### Technical contact

Technical questions on executing this protocol should be directed to and will be answered by the technical contact, Selvin Palacios (selvin.palacios@cinvestav.mx).

### Materials availability

This study did not generate new unique reagents.

### Data and code availability

This study did not generate any datasets or code.
